# Author Correction: Recent increases in tropical cyclone intensification rates

**DOI:** 10.1038/s41467-019-08963-y

**Published:** 2019-02-25

**Authors:** Kieran T. Bhatia, Gabriel A. Vecchi, Thomas R. Knutson, Hiroyuki Murakami, James Kossin, Keith W. Dixon, Carolyn E. Whitlock

**Affiliations:** 1 NOAA/Geophysical Fluid Dynamics Laboratory, Princeton, NJ 08540 USA; 2 Geosciences Department, Princeton University, Princeton, NJ 08544 USA; 3 Princeton Environmental Institute, Princeton University, Princeton, NJ 08544 USA; 4 University Corporation for Atmospheric Research, Boulder, CO 80307 USA; 5 NOAA/National Centers for Environmental Information, Center for Weather and Climate, University of Wisconsin, Madison, WI 53706 USA; 6 Engility Inc., Dover, NJ 07806 USA

Correction to: *Nature Communications* 10.1038/s41467-019-08471-z, published online 7 February 2019

The original version of this Article contained an error in the second sentence of the first paragraph of the ‘Quantile mapping’ section of the Methods, which incorrectly read ‘We primarily focus on results produced using an additive version of QDM^26^ by making use of R programming language code contained in the CRAN MBC package version 0.10–438.’ The correct version states ‘QDM^29^’ in place of ‘QDM^26^’.

Also, the third sentence of the first paragraph of the ‘Quantile mapping’ section of the Methods originally incorrectly read ‘As reported in Appendix A of Cannon et al.^26^’ the additive version of QDM is functionally very similar to the equidistant CDF matching algorithm of Li et al.^39^. The correct version states ‘Cannon et al.^29^’ in place of ‘Cannon et al.^26^’.

This has been corrected in both the PDF and HTML versions of the Article.

