# Author Correction: Recent increases in tropical cyclone intensification rates

**DOI:** 10.1038/s41467-019-11922-2

**Published:** 2019-08-28

**Authors:** Kieran T. Bhatia, Gabriel A. Vecchi, Thomas R. Knutson, Hiroyuki Murakami, James Kossin, Keith W. Dixon, Carolyn E. Whitlock

**Affiliations:** 1 NOAA/Geophysical Fluid Dynamics Laboratory, Princeton, NJ 08540 USA; 2 Geosciences Department, Princeton University, Princeton, NJ 08544 USA; 3 Princeton Environmental Institute, Princeton University, Princeton, NJ 08544 USA; 4 University Corporation for Atmospheric Research, Boulder, CO 80307 USA; 5 NOAA/National Centers for Environmental Information, Center for Weather and Climate, University of Wisconsin, Madison, WI 53706 USA; 6 Engility Inc., Dover, NJ 07806 USA

Correction to: *Nature Communications* 10.1038/s41467-019-08471-z, published online 7 February 2019.

The original version of this Article contained an error in the third sentence of the Acknowledgements, which incorrectly read ‘Kieran Bhatia and Gabriel Vecchi were supported by National Science Foundation under Grant AGS-1262099 and in part by the Carbon Mitigation Initiative at Princeton University BP International 02085(7)’. The correct version states ‘NSF EAR-1520683’ in place of ‘AGS-1262099’. Also, the original version of the Acknowledgements omitted the following from the end: ‘Any opinions, findings, and conclusions or recommendations expressed in this material are those of the authors and do not necessarily reflect the views of the National Science Foundation’. This has been corrected in both the PDF and HTML versions of the Article.

