# Supplementary material for: Recent increases in tropical cyclone intensification rates
Source: Nat Commun. 2019 Feb 7;10:635. doi: 10.1038/s41467-019-08471-z (PMC6367364; doi:10.1038/s41467-019-08471-z)
Supplement: Supplementary file 1 — Supplementary Information [file 41467_2019_8471_MOESM1_ESM.pdf]

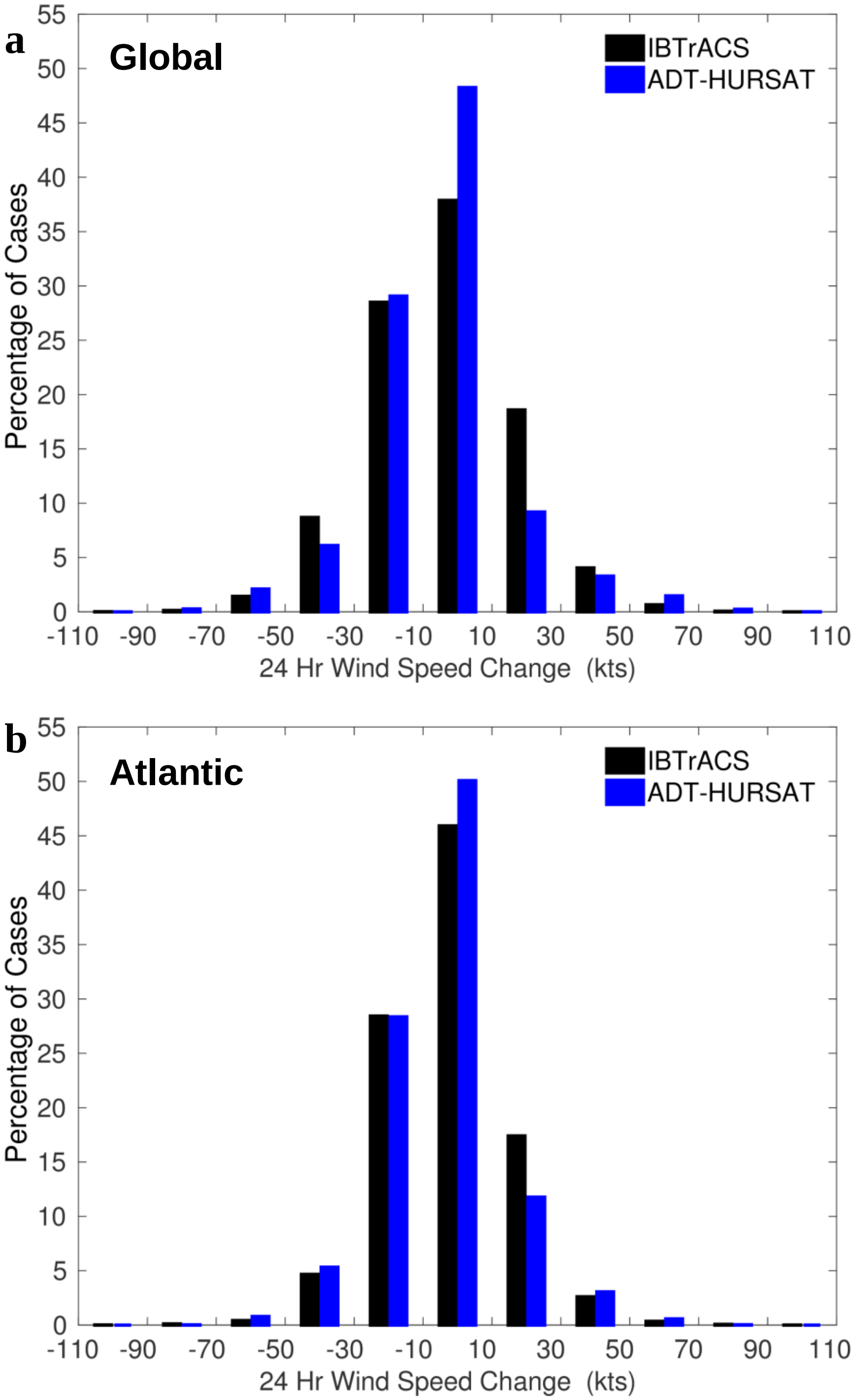

Supplementary Figure 1

a-b Percentage of all 24-hour intensity changes in each 20-knot bin calculated from IBTrACS (black) and ADT-HURSAT (blue). (a) Global and (b) Atlantic basin data from the period 1982-2009 are used to generate the histograms.

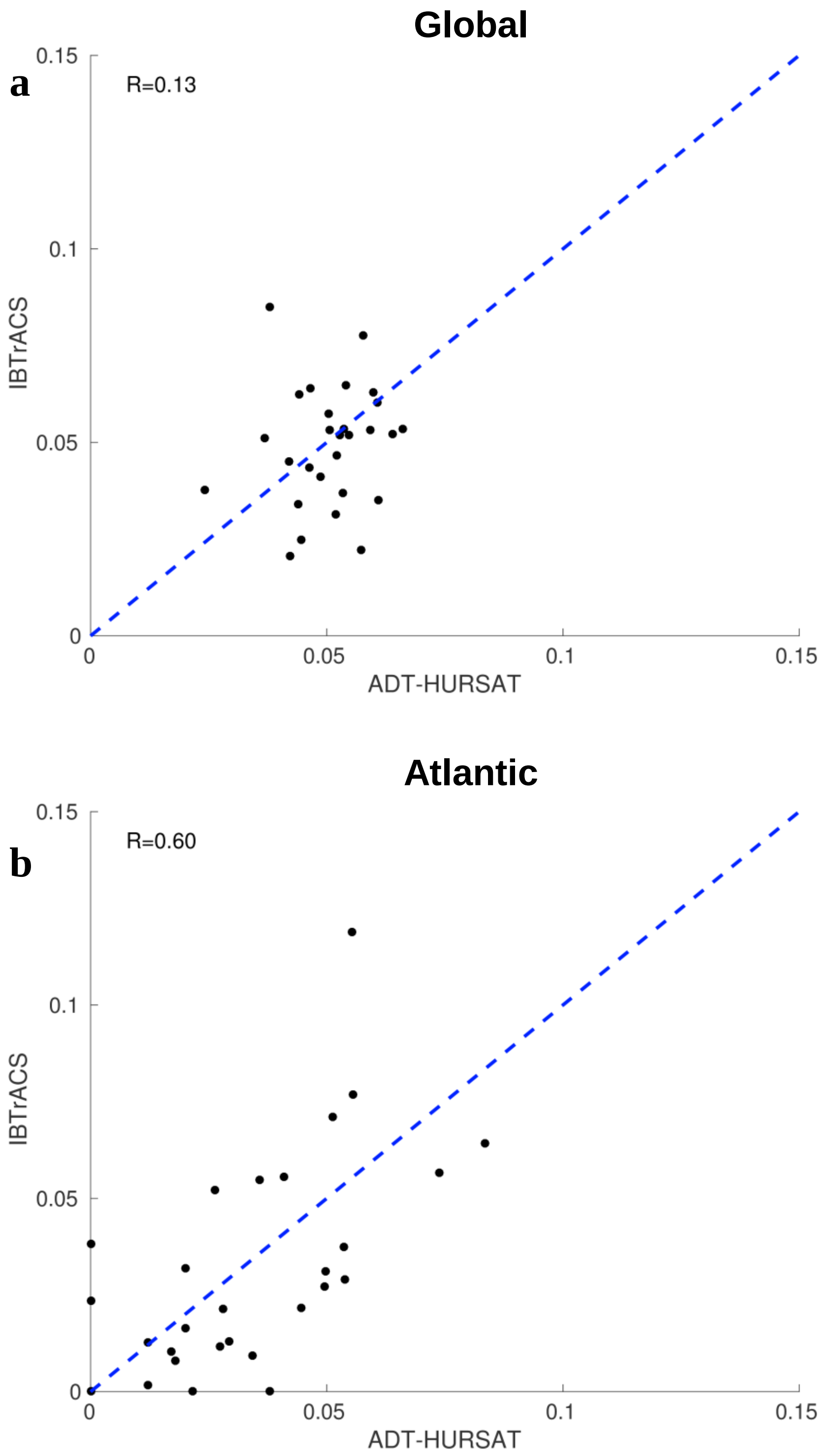

Supplementary Figure 2

a-b Annual RI ratio in ADT-HURSAT plotted against the annual RI ratio in IBTrACS for each year between 1982-2009. (a) Global and (b) Atlantic basin data are used to generate the plots. Each dot represents one year of data. The blue dashed line represents the 1 to 1 line. The

correlation coefficient for the least squares regression line is listed in the top left corner of each graph.

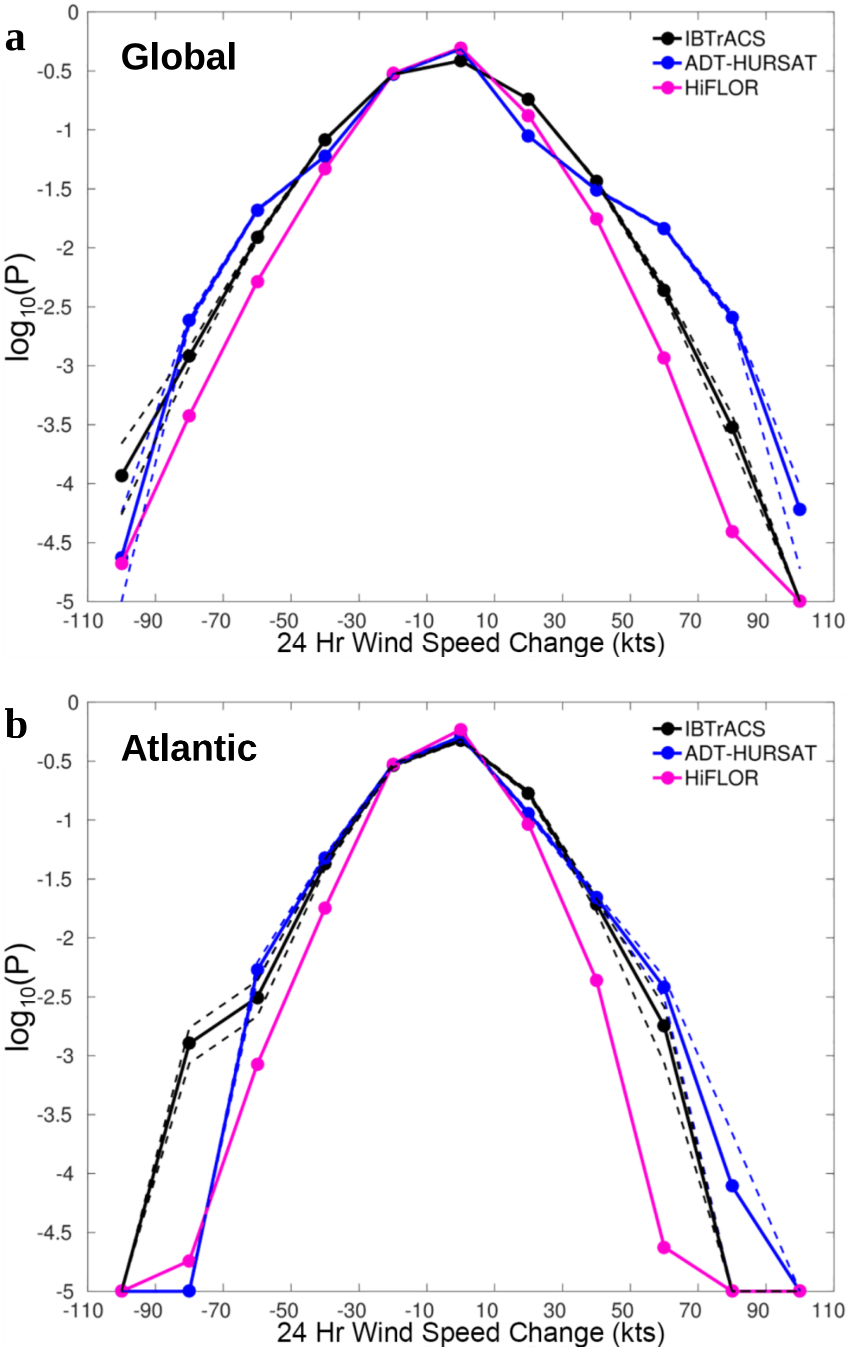

Supplementary Figure 3

a-b Common logarithm of the probability densities calculated from IBTrACS (black), ADT-HURSAT (blue), and HiFLOR 1990CTL 24-hour intensity changes. (**a**) Global and (**b**) Atlantic basin results are plotted. ADT-HURSAT and IBTrACS probabilities are computed using the 17-year period centered around 1990 (1982-1998). HiFLOR probability density curves are generated

using 250 years of intensity changes from the 1990CTL. Data is binned in 20 knot increments between -110 and 110 knots, and each bin entry is plotted as a dot on a curve. All distributions are bounded below by 10^{-5}. The dashed lines indicate the 5th and 95th percentiles of the data which are calculated using 1000 samples of the observed data perturbed with random noise (Methods).

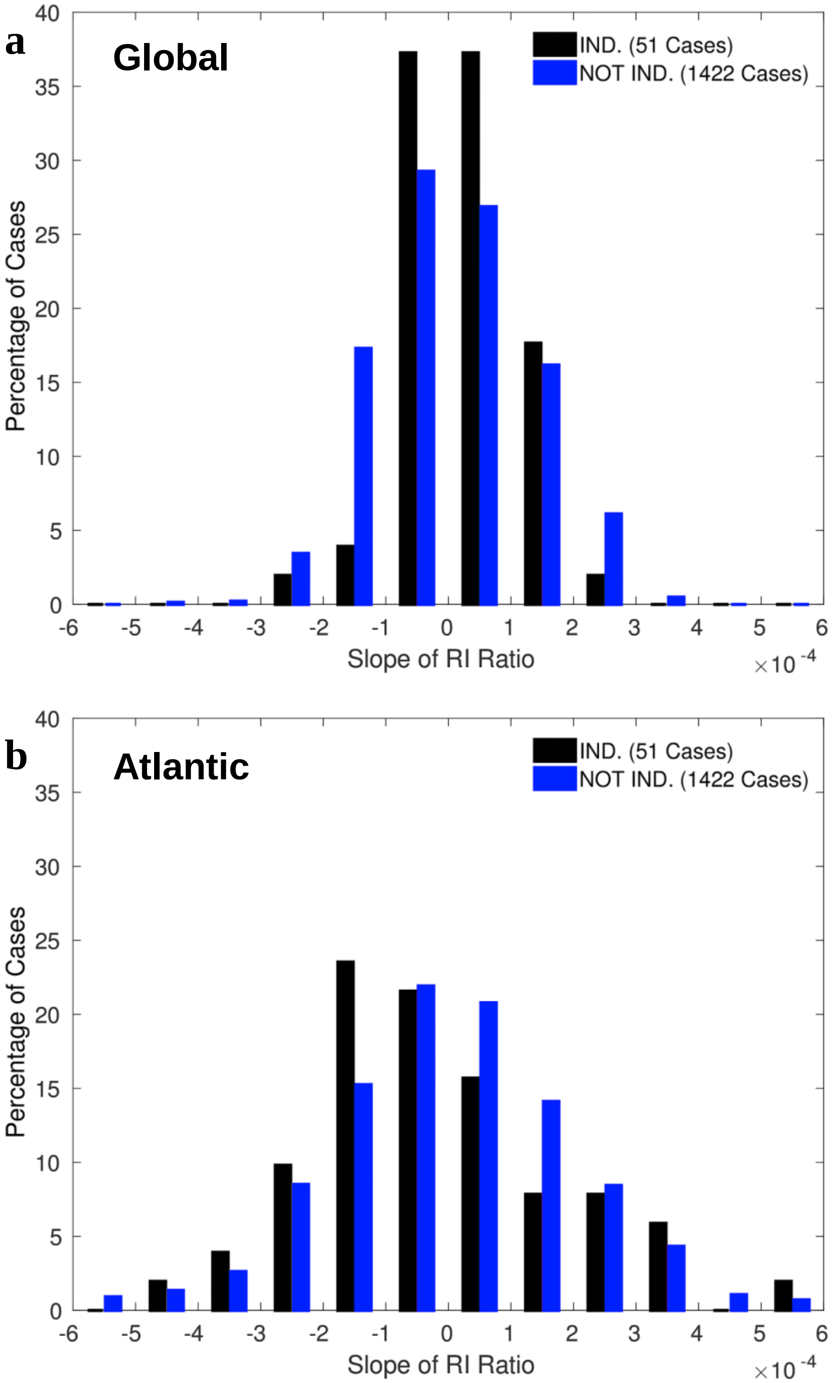

Supplementary Figure 4

a-b The probability density of RI ratio slopes that are calculated from HiFLOR 1860CTL using independent (green) and not independent (red) 28-year samples. Histograms are plotted for (a)

global and (b) Atlantic basin data. Probability densities are multiplied by 100 to obtain the percent of all RI ratio slopes that fall in each 10^{-4} bin.

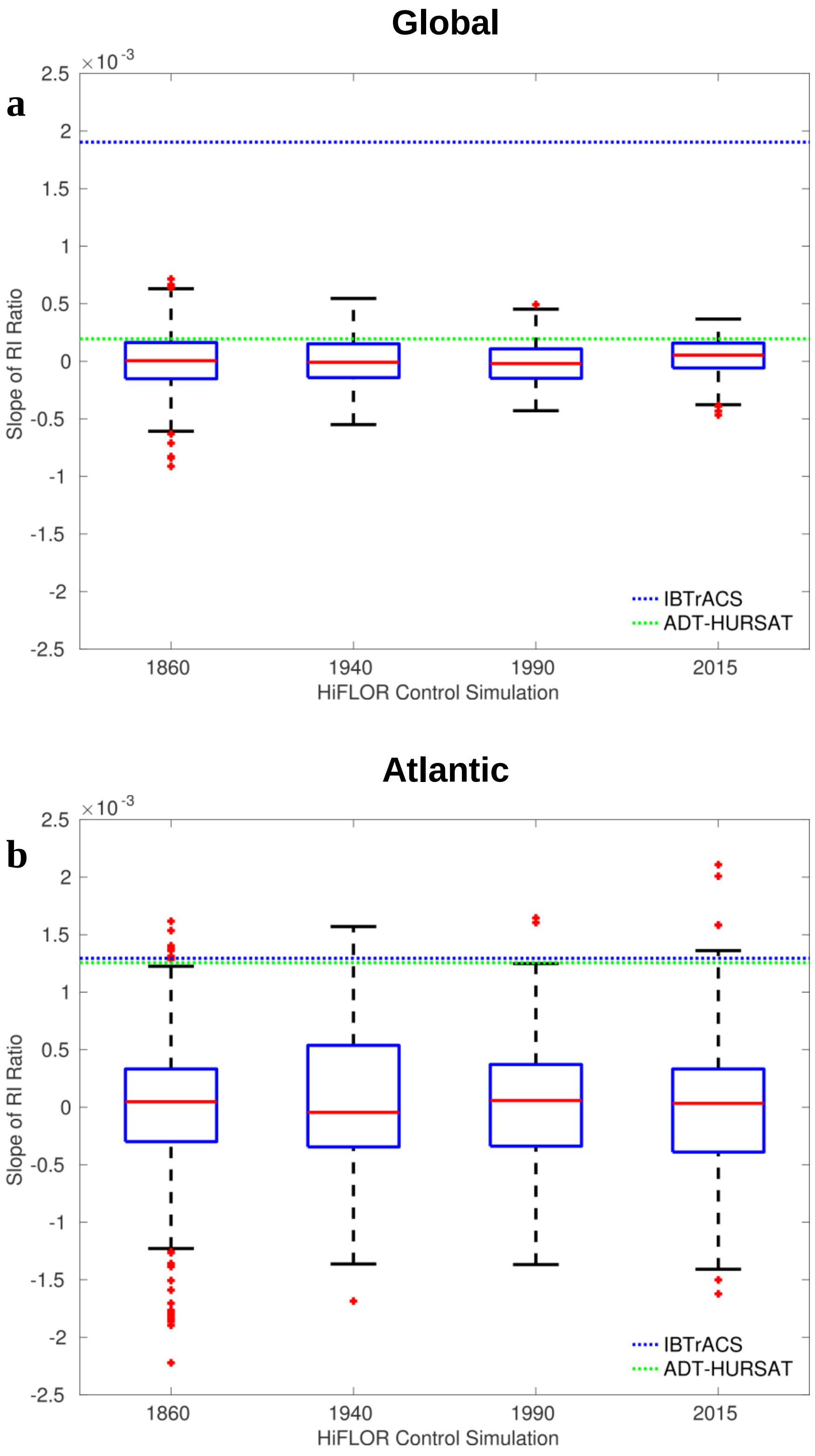

Supplementary Figure 5

a-b Box and whisker plots represent the distribution of slopes of RI ratio in each of the QDM-corrected HiFLOR control simulations. (a) Global and (b) Atlantic basin results are plotted. Each

slope is calculated by applying least squares regression analysis to annual RI ratio values in overlapping 28-year periods. Thus, the number of slopes for a control simulation is the number of available years subtracted by 28 (i.e. 1860CTL has 1422 slopes). The red line in each box indicates the median of the slope. The box is bounded by the 25th and 75th percentiles of the data, and the whiskers bracket approximately 99% of the data. Red plus signs indicate outliers whose values are outside of whiskers' range. IBTrACS and ADT-HURSAT trends in annual mean RI ratio between 1982-2009 are respectively represented by blue and green dotted lines.

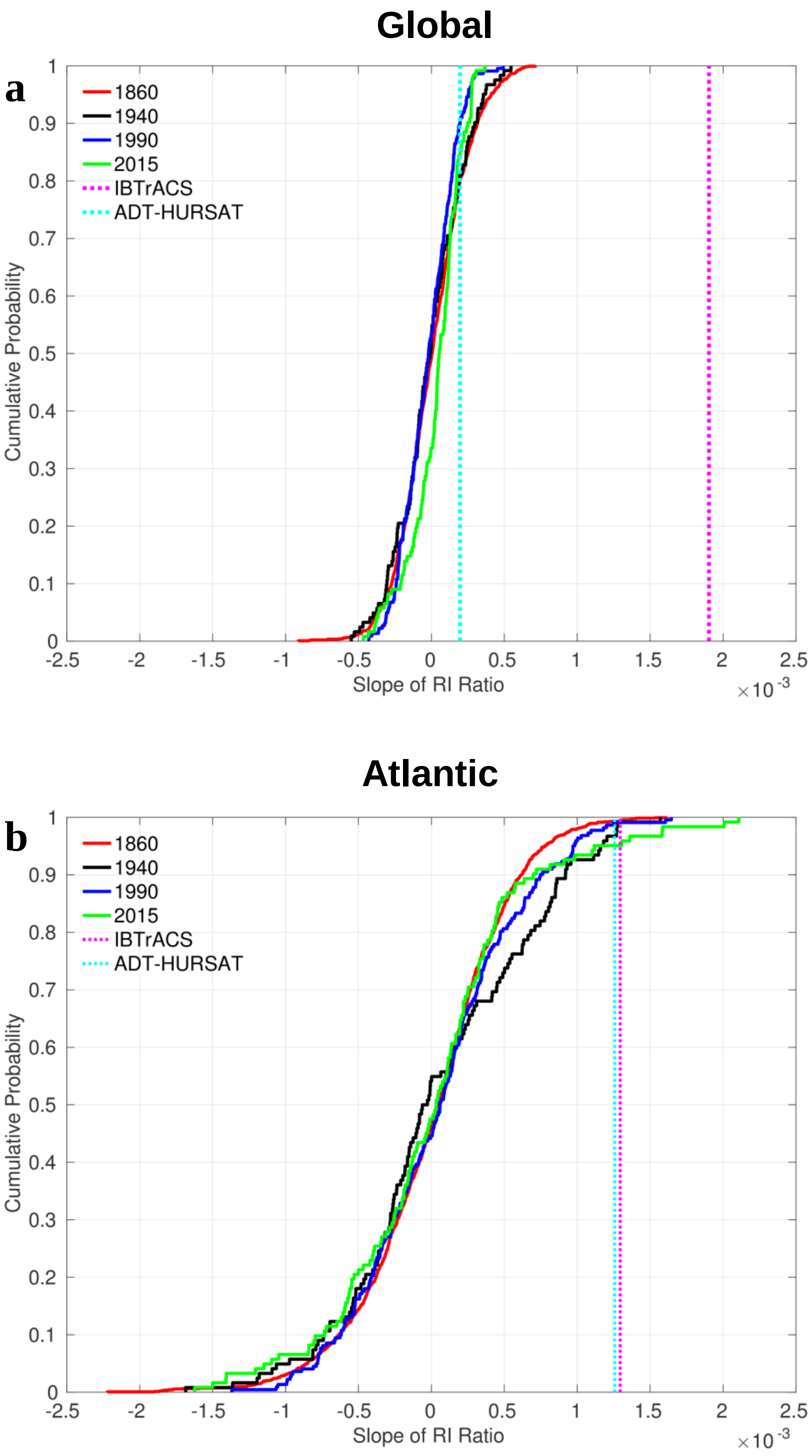

Supplementary Figure 6

a-b Cumulative Density Functions of RI ratio slopes in each of the QDM-corrected HiFLOR control simulations. (a) Global and (b) Atlantic basin results are plotted. Each slope is calculated by applying least squares regression analysis to annual RI ratio values in overlapping 28-year

periods. IBTrACS and ADT-HURSAT trends in annual mean RI ratio between 1982-2009 are respectively represented by magenta and cyan dotted lines.
